# The clinical use of epigenetics in psychiatry: a narrative review of epigenetic mechanisms, key candidate genes, and precision psychiatry

**DOI:** 10.3389/fpsyt.2025.1671122

**Published:** 2025-10-29

**Authors:** Rachel Montel Hayes, Christopher E. Mason, John J. Miller

**Affiliations:** ^1^ Department of Physiology and Biophysics, Weill Cornell Medicine, New York, NY, United States; ^2^ The His Royal Highness (HRH) Prince Alwaleed Bin Talal Bin Abdulaziz Alsaud Institute for Computational Biomedicine, Weill Cornell Medicine, New York, NY, United States; ^3^ The Feil Family Brain and Mind Research Institute, Weill Cornell Medicine, New York, NY, United States; ^4^ WorldQuant Initiative for Quantitative Prediction, Weill Cornell Medicine, New York, NY, United States; ^5^ Brain Health, Exeter, NH, United States

**Keywords:** precision psychiatry, epigenetics, genomics, personalized medicine, psychiatric disorders

## Abstract

The etiology of psychiatric disorders is complex, involving both genetic and environmental factors with emerging evidence suggesting that epigenetic modifications, including DNA methylation, histone modifications, and non-coding RNA regulation, significantly contribute to mental health. The epigenome influences the development of psychiatric disorders and human behavior and may be considered in clinical observations. Epigenetic changes have been well-established in *BDNF*, *COMT*, *FKBP5*, *NR3C1*, *SLC6A4*, and *DRD2*, genes associated with psychiatric disorders, including schizophrenia, major depressive disorder (MDD), bipolar disorder (BP), post-traumatic stress disorder (PTSD), and autism spectrum disorder (ASD). Therefore, these epigenetic marks have the potential to be suitable biomarkers for diagnostics, as predictors of prognosis, and for the development of personalized treatments. By exploring the role of clinically relevant epigenetic genes, we review the role of the epigenome in the context of psychiatric disorders and human behavior; and we consider that these changes may be observed in the context of precision psychiatry. This review synthesizes findings from over 100 original research articles and reviews spanning a range of clinical studies. Despite promising associations, challenges in the onset of precision psychiatry, such as tissue heterogeneity, small sample sizes, and lack of replication, are likely to limit translation into clinical practice. Future research in precision psychiatry will help identify clinically actionable epigenetic biomarkers, ushering in an era of genomic medicine in psychiatry.

## 1 Introduction

Mental health is rooted in biological predispositions and experiences with genetic factors contributing to psychiatric disorders but not being able to fully explain differences in symptom onset, severity, and treatment response (1). The concept that external influences such as stress, trauma, *in-utero* exposures (medications, substances of abuse, starvation, viral exposure, toxoplasmosis), early-life adversity, social relationships, and lifestyle can alter gene expression without changing the DNA sequence and affects brain function and behavior is the basis for epigenetics (2). These changes to the DNA sequence include epigenetic mechanisms that are readily reversible spanning from DNA methylation, histone modifications to non-coding RNAs and chromatin remodeling (3, 4).

In psychiatry, epigenetics is not only useful for understanding how life experiences can leave molecular marks on the genome that shape behavior but has expanded in its use as biomarkers to being able to predict risk, diagnose or characterize disease subtypes, aid in treatment response, and predict prognosis and relapse risk (5). With the advent of precision psychiatry and personalized medicine, understanding an individual’s epigenetic profile is essential, and when used in combination with genetic factors and their environmental history can open the door to targeted interventions and aid in better treatment response (6).

This review explores the major epigenetic mechanisms including DNA methylation, histone modifications, non-coding RNAs, and chromatin remodeling, and how each mechanism can contribute to gene-environment interactions in the brain (7). By examining well studied genes (including *BDNF*, *COMT*, *FKBP5*, *NR3C1*, *SLC6A4*, and *DRD2*) implicated in psychiatry disorders, including major depressive disorder (MDD), bipolar disorder (BP), schizophrenia, post-traumatic stress disorder (PTSD), attention deficit/hyperactivity disorder (ADHD), autism spectrum disorder (ASD), obsessive compulsive disorder (OCD), alcohol use disorder (AUD), and substance use disorder (SUD), and how their expression is regulated epigenetically, we gain insight into the molecular pathways linking lifetime experiences to lifelong mental health outcomes. This review also considers how epigenetic biomarkers and precision psychiatry are reshaping the future of diagnosis, prognosis, and personalized treatment, as well as the limitations hindering the onset of precision psychiatry.

## 2 Epigenetic mechanisms in psychiatry

Epigenetic modifications are readily reversible changes that influence gene expression without altering the DNA sequence (3). These mechanisms act as molecular switches or dimmers, turning genes on or off in response to internal and external stimuli (7). In psychiatry, epigenetic mechanisms play a crucial role in regulating neural pathways that underlie emotion, cognition, stress response, and behavior; and provide a compelling framework for understanding how environmental factors such as early-life adversity, trauma, social experiences, nutrition, disease, lifestyle (physical activity and quality of sleep), and therapeutic interventions, can shape brain function and contribute to the development of mental illness (5).

### 2.1 DNA methylation

DNA methylation is the process by which DNA methyltransferases (DNMTs) catalyze the addition of a methyl group (-CH_3_) to cytosine residues on the 5-carbon of the cytosine ring in CpG dinucleotides, cytosine-rich regions often near gene promoters, between genes (referred to as intergenic regions), or in gene bodies (8). When methylation occurs within promoter regions, transcription factors are blocked from binding and gene expression is reduced or silenced (9). In psychiatry, this dynamic process where methylation is readily added or removed occurs in response to developmental cues and environmental exposures such as stress, trauma, and aging (10). Because methylation patterns are inherited through cell divisions, DNA methylation is said to affect cellular memory with gene expression states preserved across time and development (11). In the context of the epigenome, DNA methylation plays a central role in important biological processes including embryonic development, X-chromosome inactivation, imprinting, tissue-specific gene expression, and brain plasticity (12).

In MDD, hypermethylation of *BDNF* promoters, especially exon IV, reduces *BDNF* expression and impairs neuroplasticity whereas hypermethylation of the *NR3C1* promoter impairs hypothalamic-pituitary-adrenal (HPA)-axis regulation following childhood trauma (13, 14). Studies in schizophrenia show that *RELN* hypermethylation reduces reelin expression and disrupts synaptic plasticity and neuronal migration; *COMT* methylation alters dopamine metabolism; and *BDNF* methylation is associated with cognitive impairment and negative symptoms (13, 15). In BD, epigenetic signatures are often state-dependent, with methylation differing in manic and depressive states compared to euthymic states (16). Likewise, increased *BDNF* methylation is reported during mood episodes, where it correlates with symptom severity (17). In ASD, global methylation differences are observed in neuronal development genes. In *MECP2*, both mutations and methylation abnormalities interact to disrupt synaptic development (18). In addiction and substance use disorders, hypermethylation of the mu-opioid receptor (*OPRM1*) is linked to heroin and alcohol dependence, and these epigenetic changes can persist to influence relapse risk (19, 20). However, *OPRM1* is not in the discussion of this review.

### 2.2 Histone modifications

Histone modifications are chemical changes to histone proteins that influence how DNA is packaged. DNA is wrapped around histone (H) octamers (with the core units made of H2A, H2B, H3, and H4) to form nucleosomes (21). Most histone modifications occur on histone tails (mainly on H3 and H4) and by chemical modifications including acetylation, methylation, phosphorylation, ubiquitination, and sumoylation. Histone acetyltransferases (HATs) add acetyl groups to lysine residues on histone tails, a modification referred to as acetylation (22). Acetylation activates gene expression by loosening chromatin and is reversed by histone deacetylases (HDACs) in a process known as deacetylation. Histones methylation is catalyzed by histone methyltransferases, which add methyl groups to lysine or arginine residues on histone tails, and reversed by histone demethylases (23). In this context, gene expression is activated or silenced depending on the specific sites and number of methyl groups (24). Other forms of histone modifications include phosphorylation, which is often associated with DNA repair and chromatin remodeling, as well as ubiquitination and sumoylation, which influence chromatin compaction and can contribute to gene repression (25).

These epigenetic changes modulate the chromatin structure and influence transcription factor accessibility and RNA polymerase binding, often interacting with other epigenetic mechanisms like DNA methylation to synergistically promote or repress gene expression (for example, methylated DNA recruiting histone-modifying enzymes) (26). In the context of psychiatry, abnormal histone modifications have been linked to multiple psychiatric disorders. Studies in depression show decreased H2K9ac at *BDNF*, in schizophrenia where H3K27me3 is modified at *GAD1*, in PTSD with repressive marks on *FKBP5*, and in addiction where changes in H3 acetylation impact reward circuits (27). HDAC inhibitors such as valproic acid and sodium butyrate can restore normal gene expression in psychiatric conditions further validating that histone marks may serve as biomarkers or targets for personalized therapies in mood and anxiety disorders (28).

### 2.3 MicroRNA regulation

MicroRNAs (miRNAs) are small (~22 nucleotides), non-coding RNA molecules that regulate gene expression post-transcriptionally by binding to target messenger RNAs (mRNAs) and suppress translation or promote mRNA degradation (29). miRNAs are both targets and regulators of epigenetic processes since they exist within a feedback loop with DNA methylation and histone modifications (30). As epigenetic targets, miRNA genes can be silenced or activated by DNA methylation or histone modifications, and as epigenetic regulators, miRNAs can target enzymes like DNMTs, HDACs, or histone methyltransferases for silencing, thereby influencing epigenetic landscapes. miRNAs are context-dependent, so their effect may vary by cell type, developmental stage, and environment, and they are also dynamic, changing in response to stress, drugs, experiences, and hormones (31). miRNAs are abundant in the brain and have crucial roles in neuronal development and differentiation, synaptic plasticity and learning, stress responses, and emotional regulation.

Changes in miRNA expression are clinically relevant across multiple psychiatric disorders. Genome-wide association studies (GWAS) in schizophrenia have implicated miR-137 as one of the most significant risk loci for schizophrenia (32). This miRNA regulates neurodevelopment and synaptic signaling genes, such as *CACNA1C* and *TCF4*, and has been proposed as a biomarker, with therapeutic modulation offering a potential means to stabilize synaptic function (32, 33). In depression, miR-124 is abundant in the brain and regulates neurogenesis and stress response; dysregulated levels have been associated with hippocampal neuroplasticity deficits (34). Other studies have reported that plasma miR-124 levels increase after citalopram treatment relative to untreated controls (35). In similar studies, miR-16-5p is reduced in peripheral blood of individuals with MDD and BD (36). Despite this findings, a major limitation is that miRNA expression is often assessed in peripheral blood rather than the brain directly. In schizophrenia, miR-132, miR-134, miR-1271, miR-664, miR-200c, and miR-432 are significantly decreased; however, antipsychotic treatment increases miR-132, miR-664, and miR-1271 compared with pre-treatment levels (37). In other psychiatric disorders, notable miRNAs include miR-34a and miR-181, which are significantly altered in schizophrenia; miR-146a and miR-134, which are altered in ASD, and miR-121 and miR-132, which are associated with addiction (38).

### 2.4 Chromatin remodeling

Chromatin remodeling is the dynamic structural change in chromatin which regulates access to DNA for transcription, replication, and repair. Chromatin remodeling controls the transition between euchromatin and heterochromatin thereby allowing or blocking gene expression (39). This process is mediated by ATP-dependent chromatin remodeling complexes, which move, eject, or restructure nucleosomes to increase DNA accessibility. Key ATP-dependent chromatin remodeling complexes include: (1) SWI/SNF, which slides or ejects nucleosomes to open chromatin (e.g., BRG1, BAF complex); (2) ISWI, which spaces nucleosomes for transcriptional regulation (e.g., SNF2H); (3) CHD, which contributes to nucleosome assembly and remodeling (e.g., CHD1, CHD8); and (4) INO80, which couples remodeling to DNA repair and replication (e.g., INO80, SRCAP) (40). Chromatin remodeling works with other epigenetic mechanisms by directly interacting with histone modifiers, DNA methylation machinery, and non-coding RNAs (41). In the context of psychiatric and neurological disorders, mutations in chromatin complexes have been observed in schizophrenia where synaptic gene regulation is disrupted, and these mutations are also high-confidence genetic risk factors for ASD (42). In depression, altered chromatin accessibility have been observed at *BDNF*, *GAD1*, *NR3C1* in response to stress (43).

Currently, chromatin accessibility profiling (e.g., ATAC-seq) is being explored as a biomarker tool in psychiatry (44). Because most psychiatric GWAS findings lie in noncoding regions (i.e., enhancers or promoters), ATAC-seq helps identify which of these regions are open and accessible in brain cells. For example, ATAC-seq can reveal which accessible regions are specific to particular cell types, such as excitatory neurons, interneurons, or microglia. Therefore, single-cell chromatin accessibility profiling (scATAC-seq), together with gene expression assays, is used to identify disease-relevant fetal and adult brain cell types implicated in major depressive disorder, body mass index (BMI), ADHD, and schizophrenia (45). This approach has been applied to the identification of single nucleotide polymorphisms (SNPs) with schizophrenia risk in accessible enhancers of glutamatergic neurons, notably within genes such as *CACNA1C*, which plays a key role in synaptic function (46). Studies employing ATAC-seq have revealed increased accessibility of glucocorticoid-responsive enhancers in the brains of stressed individuals. These insights have driven the development of FKBP5 inhibitors, which are now being explored in clinical trials for PTSD and alcohol use disorder (47, 48).

### 2.5 Environmental influence on epigenetic changes

Environmental epigenetics studies how external factors like stress, diet, toxins, infections, relationships, and experiences, can lead to long-lasting changes in gene expression by altering the epigenome (49). For example, nutrition provides methyl donors and cofactors for epigenetic enzymes such as folate, B12, and choline which influence DNA methylation and for which undernutrition affects *IGF2* expression (50). Exercise promotes histone acetylation and *BDNF* expression with aerobic activity increasing BDNF (51). Toxins and pollutants can alter the epigenome by inducing oxidative stress and disrupting methylation machinery, and in the brain, bisphenol A (BPA), lead, and air pollution alter methylation in brain development genes (52–54). However, the evidence surrounding these external factors is less robust than that for early-life stress and warrants further investigation to become clinically actionable. In the case of early-life stress, commonly experienced through childhood abuse, DNA methylation of stress-regulating genes (i.e. *NR3C1*, *FKBP5*) increases, hypermethylating the glucocorticoid receptor and altering the HPA axis (10). This effect stems into social environments that affect methylation of social, stress, and emotion-related genes; and, in this context, studies demonstrate that having a high-quality rearing lowers *OXTR* and *NR3C1* methylation (55). Despite these modifications, maladaptive epigenetic changes can be reversed or normalized by psychological interventions such as cognitive behavioral therapy (CBT) and mindfulness, and studies have examined the potential of these interventions to reduce methylation of *FKBP5* and *SLC6A4* and for psychiatric medications like SSRIs, antipsychotics and the HDAC inhibitor valproate to alter epigenetic landscapes (56, 57).

## 3 Clinically relevant epigenetically regulated genes in psychiatry

### 3.1 Brain-derived neurotrophic factor

The brain-derived neurotrophic factor (BDNF) is an essential protein in the brain and nervous system. BDNF is a growth factor that supports neuroplasticity, neuronal survival, neurogenesis, and contributes to neural repair and recovery after brain injuries like stroke or trauma (58). *BDNF* is highly expressed throughout the brain including the hippocampus, prefrontal cortex, amygdala, cortex, and basal forebrain (59). In the context of cognitive function, higher BDNF levels are associated with improved cognitive performance including memory, attention, and problem-solving (60). In mood regulation, low BDNF levels are linked to depression and anxiety. The *BDNF* gene has multiple promoters which are prone to epigenetic regulation. For example, exon IV of *BDNF* is highly sensitive to environmental signals and hypermethylation of *BDNF* promoter regions, especially exon IV, reduces *BDNF* expression (61).

Studies have reported reduced *BDNF* expression in people with MDD, PTSD, schizophrenia, and after early-life stress or childhood trauma (62). In depression, studies report low peripheral BDNF levels and higher promoter methylation, while antidepressants and electroconvulsive therapy (ECT) increase *BDNF* expression and reverse methylation (63, 64). In BD, *BDNF* epigenetic changes are state-dependent, with lower levels during manic and depressive episodes compared with euthymia (65). Studies in PTSD and suicidality demonstrate that early-life trauma is associated with long-lasting *BDNF* promoter methylation, affecting stress reactivity and fear extinction, and for suicide victims, *BDNF* promoter hypermethylation is observed in the prefrontal cortex and hippocampus (66–68).

*BDNF* is subject to other forms of epigenetic regulation, including histone modifications, whereby acetylation enhances gene expression and histone deacetylation represses expression (69). Selective serotonin reuptake inhibitors (SSRIs) and HDAC inhibitors can increase histone acetylation at the *BDNF* loci therefore boosting expressions (70). In addition to DNA and histone modifications, *BDNF* expression is epigenetically regulated by miRNAs (including miR-132 and miR-206), which can bind *BDNF* mRNA and suppress translation. Dysregulation of these miRNAs is linked to mood disorder and schizophrenia (71)Although important progress has been made, notable limitations remain. The epigenetic regulation of *BDNF* varies by isoform and promoter, but this distinction is often overlooked, and most studies are further limited by the absence of rigorously controlled longitudinal cohorts. A summary of these epigenetic alterations has been captured in **Table 1**.

### 3.2 Catechol-O-methyltransferase

The catechol-O-methyltransferase (COMT) enzyme metabolizes catecholamines including the neurotransmitters dopamine, norepinephrine, and epinephrine by transferring a methyl group from S-adenosylmethionine (SAM) to these catecholamines thus facilitating catecholamine degradation and regulating their levels in various tissues including the brain (72). COMT exists in two primary forms: soluble COMT (S-COMT) and membrane-bound COMT (MB-COMT). S-COMT is predominantly found in peripheral tissues like the liver, kidneys, and blood and plays a critical role in metabolizing catecholamines circulating in the body. MB-COMT is mainly produced by nerve cells in the brain and is integral to the degradation of neurotransmitters within the central nervous system (73). In the brain, COMT regulates dopamine levels in regions crucial for cognitive functions like the prefrontal cortex. Based on the concept that COMT regulates dopamine metabolism, epigenetic regulation of *COMT* influences not only gene expression and enzyme activity but neurotransmitter metabolism and alters dopamine signaling, key features in schizophrenia and cognitive dysfunction (74). In fact, methylation of the *COMT* promoter has been associated with reduced gene expression in both brain and peripheral tissues, leading to increased dopamine availability in the prefrontal cortex (75).

Studies in schizophrenia support that hypomethylation of the *COMT* promoter increases COMT activity and enhances dopamine degradation in the prefrontal cortex. This dopamine deficit could contribute to the cognitive impairment and negative symptoms which are 2 of the three main sub-syndromes in individuals with schizophrenia (76). In BD studies, dysregulation of dopamine metabolism may contribute to the signature mood instability of this disorder (77). Studies in depression show that hypermethylation of *COMT* is associated with MDD, and often correlated with stress exposure and treatment response (78). In PTSD, increased *COMT* methylation is associated with impaired fear inhibition, which affects stress responsivity via dopamine regulation (79). In the context of environmental interactions, cigarette smoking is associated with hypermethylation of the MB-COMT promoter, while cannabis use interacts with the *COMT* Val158Met genotype/epigenotype to influence psychosis risk (80, 81). Although less well characterized, histone modifications also affect *COMT* expression, and the general mechanisms such as histone acetylation and methylation are known to influence gene expression in neuropsychiatric contexts (82). Several limitations remain in these studies, notably the requirement for larger cohorts, independent study replication, and efforts to disentangle genotype-epigenotype interactions, such as demonstrating how Val/Met allele effects differ according to methylation status. A summary of these epigenetic alterations has been captured in **Table 1**.

**Table 1:** Epigenetic alterations across psychiatric disorders.

Disease state	Epigenetic change(s)
Major Depressive Disorder (MDD)	*BDNF*: reduced peripheral levels; promoter hypermethylation; reversed by antidepressants & electroconvulsive therapy
	*COMT*: hypermethylation associated with stress & treatment response
	*FKBP5*: elevated expression via demethylation; linked to altered brain structure/function
	*NR3C1*: hypermethylation with childhood trauma decreases GR expression & HPA axis dysregulation
	*SLC6A4*: promoter hypermethylation (especially S allele carriers) reduces SERT expression; altered treatment response to SSRIs/psychotherapy
Bipolar Disorder (BD)	*BDNF*: state-dependent changes; lower levels during manic/depressive episodes vs. euthymia
	*COMT*: dysregulated dopamine metabolism may drive mood instability
	*FKBP5*: methylation variation suggests role in mood regulation/stress response
	*NR3C1*: altered methylation patterns contribute to neuroendocrine dysfunction (variable findings)
Post-Traumatic Stress Disorder (PTSD)	*BDNF*: long-lasting promoter hypermethylation after trauma; decreased stress reactivity/fear extinction
	*FKBP5*: trauma + risk allele carriers have altered methylation; increased risk of PTSD
	*NR3C1*: hypermethylation from early trauma leads to decreased GR function, sustained stress reactivity/fear memory consolidation
	*SLC6A4*: hypermethylation in S allele carriers with trauma exposure have increased PTSD symptoms
Schizophrenia	*BDNF*: reduced expression linked to cognitive impairment & negative symptoms
	COMT: hypomethylation increases COMT activity leading to excess dopamine degradation in prefrontal cortex and cognitive/negative symptoms
	*NR3C1*: altered methylation patterns contribute to neuroendocrine dysfunction (variable findings)
	*DRD2*: altered promoter methylation in prefrontal cortex & striatum; dysregulated dopamine signaling
	*miRNAs*: decreased miR-132, miR-134, miR-1271, miR-664, miR-200c, miR-432; some increase after antipsychotic treatment
Suicidality	*BDNF*: promoter hypermethylation in prefrontal cortex & hippocampus of suicide victims
	*NR3C1*: hypermethylation in postmortem brain tissue from abuse victims causes trauma embedding
	*SLC6A4*: increased methylation in individuals with suicide attempts/ideation after trauma
Anxiety Disorders	*NR3C1*: epigenetic dysregulation of GR signaling increases cortisol responsiveness
	*SLC6A4*: elevated methylation associated with GAD, panic disorder, social anxiety, amygdala hyperreactivity
Autism Spectrum Disorder (ASD)	Global methylation differences in neuronal development genes (e.g., *MECP2*)
	Altered miRNAs (miR-146a, miR-134)
Obsessive-Compulsive Disorder (OCD)	*FKBP5*: reduced methylation at intron 7 CpG site in men causes possible HPA axis dysfunction
Addiction & Substance Use Disorders	*OPRM1*: hypermethylation linked to heroin & alcohol dependence; persistent changes affect relapse risk
	*DRD2*: hypermethylation decreases expression causing reward deficiency & addiction vulnerability
	*miRNAs*: altered miR-121 and miR-132
Attention-Deficit/Hyperactivity Disorder (ADHD)	*SLC6A4*: mixed findings linking methylation differences to neurodevelopmental risk
	*DRD2*: polymorphisms and methylation status associated with impulsivity & dopamine dysfunction

### 3.3 FK506 binding protein 5

The FK506 Binding Protein 5 (*FKBP5*) gene encodes FKBP51, a co-chaperone protein that modulates the glucocorticoid receptor (GR) complex and affects cortisol signaling (83). By altering GR sensitivity, *FKBP5* plays a critical role in stress hormone regulation, influencing the HPA axis, and impacting various psychiatric conditions (84). Stress exposure such as early-life stress decreases methylation of *FKBP5* and upregulates *FKBP5* gene expression which reduces GR sensitivity and may cause an exaggerated stress response (85). In an interplay between the genome and epigenome, polymorphisms in the *FKBP5* gene (such as rs1360780) are linked to differential methylation patterns in response to stress that influence gene expression and stress reactivity (56).

In individuals with PTSD, carriers of *FKBP5* polymorphisms who experience childhood trauma exhibit altered *FKBP5* methylation, which increases their risk of developing the disorder (86). Stress has also been shown to influence chromatin accessibility at the *FKBP5* locus, although this area remains less well characterized than DNA methylation (87). Additionally, microRNAs such as miR-15 and miR-511 have been proposed to regulate *FKBP5* expression, linking it to pathways of inflammation and stress response (88). MDD studies show that *FKBP5* expression is epigenetically elevated and associated with structural and functional alterations in brain regions involved in emotional processing further contributing to MDD pathophysiology (89). With BD, methylation variations in *FKBP5* expression are observed which suggests a role in mood regulation and stress response mechanisms (90). In OCD, reduced DNA methylation at the *FKBP5* intron 7 CpG site has been reported in male patients relative to controls. While these findings suggest a potential role for *FKBP5* methylation in the pathogenesis of OCD, additional studies are required to confirm whether altered DNA methylation at intron 7 contributes to disease mechanisms in concert with HPA axis dysregulation (91). Therapeutic strategies shown to normalize *FKBP5* methylation include antidepressants, cognitive behavioral therapy (CBT), and mindfulness practices (92–94). Importantly, *FKBP5* alterations are not observed in all trauma survivors, underscoring limitations in biomarker specificity and sensitivity that warrant further investigation. A summary of these epigenetic alterations has been captured in **Table 1**.

### 3.4 Glucocorticoid receptor

The *NR3C1* gene encodes the glucocorticoid receptor (GR), the main receptor for cortisol and a key regulator of the HPA axis (95). As the body’s primary stress hormone, cortisol binds to GR allowing GR to translocate to the nucleus and regulate the expression of stress-responsive genes. GR signaling is critical for regulating the HPA axis feedback loop and controls inflammation, immune responses, and metabolism while modulating neurodevelopment, mood, and cognitive function (96). Based on the concept that *NR3C1* is critical for glucocorticoid signaling and stress regulation, hypermethylation of *NR3C1* in individuals exposed to childhood adversity correlates with increased vulnerability to PTSD, depression, and suicide risk (2). Targeting *NR3C1* methylation patterns may provide therapeutic potential for stress-related psychiatric disorders (97).

The *NR3C1* gene is said to be environmentally programmed by stress reactivity because early-life adversity such as childhood abuse, neglect, and maternal stress are strongly associated with hypermethylation of exon 1F. The *NR3C1* exon 1F is a critical promoter region that undergoes epigenetic regulation via DNA methylation where hypermethylation of this region reduces *NR3C1* expression, decreases GR availability, impairs cortisol feedback sensitivity, and heightens or prolongs stress responses (97). In MDD, hypermethylation of *NR3C1* promoter regions in individuals with childhood trauma is associated with reduced GR expression and dysregulation of the HPA axis, a hallmark of depression (14). PTSD studies show *NR3C1* hypermethylation from early trauma leads to decreased GR function, sustained stress reactivity, and fear memory consolidation (56). In studies of post-mortem brain tissue from suicide completers with a history of abuse, *NR3C1* methylation is increased, suggesting a biological embedding of trauma at the molecular level (43). Several studies on anxiety disorders link epigenetic dysregulation of GR signaling with generalized anxiety and panic disorder, likely through heightened cortisol responsiveness (98). *NR3C1* methylation patterns are also altered in schizophrenia and BD, which may contribute to neuroendocrine dysfunction in these disorders, although these findings are more variable (99).

### 3.5 Serotonin transporter (*SLC6A4*/5-HTTLPR)

The *SLC6A4* gene encodes the serotonin transporter (SERT), a membrane protein responsible for the reuptake of serotonin (5-HT) from the synaptic cleft back into presynaptic neurons (100). *SLC6A4* regulates serotonin availability in the brain, impacts mood, emotion, anxiety, and stress regulation, and is the main target of SSRIs used in treating MDD and anxiety disorders (101). The serotonin-transporter-linked polymorphic region (5-HTTLPR) is a region in the *SLC6A4* promoter with two main alleles: the short (s) allele and the long (l) allele. The short allele has lower transcriptional efficiency and reduced SERT expression and is often associated with increased emotional reactivity and greater susceptibility to stress whereas the long allele has higher transcription and increased SERT expression (102). *SLC6A4* is epigenetically regulated by DNA methylation influencing *SLC6A4* expression independently or interactively with 5-HTTLPR genotype (103). Methylation of CpG sites in the *SLC6A4* promoter, especially near exon 1, and the 5-HTTLPR region reduces *SLC6A4* expression lowering SERT expression, SERT availability, and increasing extracellular serotonin. This effect is often independent of genotype and can result from environmental exposures such as early-life trauma or stress. S allele carriers that experience early-life stress or trauma tend to show higher *SLC6A4* promoter methylation suggesting that stress programs serotonin signaling via epigenetic changes (104). Childhood adversity, maternal depression, or prenatal stress increase *SLC6A4* methylation and cause impaired stress resilience and altered emotional regulation later in life (105).

*SLC6A4* hypermethylation, together with the presence of the S allele, is linked to an increased risk of depression because SERT expression and serotonin uptake are reduced, especially in those with childhood adversity (105). SSRIs may normalize some of these methylation changes and are often examined within gene x environment (GxE) models in psychiatric research, which also suggests that *SLC6A4* methylation can be used as an indicator of treatment response to SSRIs or psychotherapy (106). Elevated *SLC6A4* methylation is also associated with generalized anxiety disorder (GAD) and panic disorder, correlates with increased anxiety traits, social anxiety, and amygdala hyperreactivity, and may explain how altered serotonin signaling affects fear processing and threat detection (107). With PTSD, *SLC6A4* methylation is increased in S allele carriers with trauma exposure. Several studies in veterans and trauma survivors show higher methylation at *SLC6A4* CpGs in patients with PTSD symptoms, demonstrating that these epigenetic changes reflect persistent alterations in stress reactivity pathways and can serve as biomarkers of stress vulnerability (105). In suicide studies, individuals with a history of suicide attempts or ideation that have been exposed to trauma have increased *SLC6A4* methylation (43). Some studies link neurodevelopmental risk for attention deficit hyperactivity disorder (ADHD) to differences in *SLC6A4* methylation patterns although these findings are mixed (108). A summary of these epigenetic alterations has been captured in **Table 1**.

### 3.6 Dopamine Receptor D2

The *DRD2* gene encodes the D2 subtype of the five dopamine receptors. As a G-protein-coupled receptor (GPCR), DRD2 is involved in modulating dopaminergic neurotransmission and plays a role as an inhibitory receptor by reducing neuronal excitability and dopamine synthesis via Gi/o protein coupling (109, 110). *DRD2* expression is observed at high levels in the striatum, prefrontal cortex, and limbic regions to regulate reward processing, motivation and reinforcement, cognitive control, movement and motor coordination (111). There are two main D2 receptor isoforms that are synthesized by differential splicing of introns and exons form the D2 gene. The short isoform (D2S) is a presynaptic auto-receptor on dopamine neurons that regulates dopamine release, and the long isoform (D2L) is a postsynaptic receptor involved in postsynaptic signaling (112).

*DRD2* is subject to epigenetic regulation and changes in *DRD2* expression may impact dopamine signaling and contribute to neuropsychiatric disorders (113). For example, DNA hypermethylation of *DRD2* promoter regions, such as in exon 1 or upstream CpG islands, downregulates receptor expression; on the contrary, hypomethylation increases *DRD2* levels thereby altering dopamine sensitivity (114). Histone deacetylase inhibitors (HDACi) can increase *DRD2* expression in certain contexts, suggesting chromatin remodeling as a DRD2 epigenetic regulator (28). MicroRNAs, such as miR-9 and miR-326, are another example of *DRD2* epigenetic regulation since they target *DRD2* mRNA and reduce translation. Dysregulation of these miRNAs is linked to changes in *DRD2* signaling in psychiatric illness (115).

Postsynaptic D2 receptor overactivation, resulting from increased presynaptic dopamine release in the nucleus accumbens (located in the ventral striatum), is central to the dopamine hypothesis of schizophrenia (116). Postmortem studies show altered *DRD2* methylation in the prefrontal cortex and striatum of individuals with schizophrenia (117). Studies in substance use disorders including cocaine, alcohol, and nicotine show that substance use can alter *DRD2* methylation in animal and human models. In this context, reduced *DRD2* expression from DNA hypermethylation is associated with reward deficiency and addiction vulnerability (118). In ADHD, *DRD2* polymorphisms and methylation status are linked to impulsivity and dopaminergic dysfunction (119). A summary of these epigenetic alterations has been captured in **Table 1**.

## 4 Clinical implications of epigenetics in psychiatry

### 4.1 Epigenetic-based therapies

Epigenetic-based therapies are designed to regulate gene expression by targeting epigenomic processes while leaving DNA sequence itself unchanged. An example of an epigenetic-based therapy is HDAC inhibition, which increase histone acetylation thereby relaxing the chromatic and increasing gene expression (27). Valproic acid, sodium butyrate, and Vorinostat (SAHA) are examples of HDAC inhibitors that are currently used to target depression, schizophrenia, BD, PTSD and are being preclinically explored for ASD (120). Several therapies are in experimental stages including bromodomain and extra-terminal domain (BET) inhibitors, which bind to BET proteins and prevent them from interacting with acetylated histones modulating gene transcription. JQ1 is an investigational BET inhibitor for addiction, mood disorders, and preclinical neuroinflammation (121).

In addition to HDAC inhibitors, several experimental miRNA-based therapies for psychiatric disorders like schizophrenia, MDD, anxiety, and neurodevelopmental disorders are currently under investigation (122). These miRNA-based therapies include miR-124, miR-132, miR-135 and miR-146a (123). Other epigenetic-based therapies include epigenetic editing approaches using CRISPR/dCas9 for targeted DNA methylation or demethylation, histone editing by CRISPR-dCas9-TET1 for demethylation, and CRISPR-dCas9-HDAC for repression. These therapies focus on reversing stress-induced gene silencing, and studies demonstrate that such interventions can alleviate symptoms of MDD, anxiety, PTSD, while enhancing cognitive resilience (124). They are currently being evaluated in preclinical models of PTSD, addiction, and MDD (125, 126).

### 4.2 Precision psychiatry and future directions

In the era of precision psychiatry, understanding epigenetic patterns can refine diagnosis and improve clinical subtyping. For example, methylation of *NR3C1*, *BDNF*, and *FKBP5* can differentiate subtypes of MDD, PTSD, or schizophrenia, help to identify stress-responsiveness compared with neurodevelopmental forms of illness, and be used as predictors of risk and resilience (5). Epigenetic markers like *SLC6A4* or *OXTR* methylation can predict risk for MDD or anxiety after trauma and individual resilience profiles (127). This epigenetic information may be useful for detecting at-risk individuals such as trauma-exposed youth and providing early intervention (128). In the context of pharmaco-epigenetics, methylation of genes like *BDNF*, *SLC6A4*, and *FKBP5* may predict response to SSRIs, psychotherapy, or electroconvulsive therapy (129). For example, clinical studies suggest that low *BDNF* methylation correlates with better SSRI response and high *NR3C1* methylation predicts poorer CBT response in PTSD (56). Additionally, epigenetic biomarkers could be used to guide novel epigenetic therapies in patients with hypermethylated *GAD1* or *RELN* who may benefit from HDAC inhibitors or DNMT inhibitors to restore expression of key neuroplasticity genes (130).

## 5 Limitations

Several limitations hinder the application of epigenetic findings as biomarkers in psychiatry. First, access to brain tissue is limited, and sampling relies on that from blood, saliva, or postmortem samples, which may not reflect central nervous system changes. Cell type specificity varies since epigenetic marks vary dramatically between neurons, glia, and other brain cells (45). Therefore, bulk tissue analyses can obscure important differences at the cellular level. As discussed, epigenetic modifications are dynamic and can change rapidly. Therefore, stress, diet, and/or medications allow for temporal variability, which may not be reflected in the long-term pattern. Different assays can yield inconsistent results. For example, bisulfite sequencing vs. ChIP-seq (chromatin immunoprecipitation followed by sequencing) can complicate reproducibility across studies. Lastly, most large-scale genome and epigenome reference datasets, including those used for methylation and chromatin analyses, are derived predominantly from individuals of European ancestry. Therefore, the current body of research lacks ancestral diversity among study populations and may obscure ancestry-specific epigenetic signatures or gene-environment interactions that contribute to psychiatric risk. Future research efforts should focus on expanding cohort diversity to improve the equity, accuracy, and translational relevance of epigenetic discoveries.

In addition to these limitations, there are several confounding effects, including lifestyle, socioeconomic factors, substance use, and comorbid medical conditions, that need to be considered, as they can shape the epigenome, making it difficult to isolate psychiatry-specific signatures. It is important to emphasize that epigenetic findings have not yet been validated for routine use in psychiatric practice. Lastly, because psychiatric disorders are influenced by thousands of genetic and environmental factors, an exclusive focus on epigenetics risks oversimplifying the complexity of disease etiology.

## 6 Conclusion

In this review, we have outlined the epigenetic mechanisms implicated in psychiatric disorders and their relation to mental health conditions. Extensive studies demonstrate that epigenetic modifications influence the development and progression of psychiatric disorders as well as human behavior. With ongoing research into the clinical applications of epigenetically informed interventions, it is plausible that such treatments will become part of routine psychiatric care. As highlighted, the transition toward precision psychiatry requires overcoming several limitations. Key challenges include the need for standardization across cell type specificity and assay protocols, rigorous control of confounding factors that may bias epigenetic findings, and integration of epigenetic data within the broader context of gene-environment interactions and systems biology. Future research should include large, longitudinal cohorts, tissue-specific analyses, and multi-omics integration, considering not only genomics but transcriptomics and proteomics as well. As investigation into epigenetic biomarkers continues to progress, and the understanding of the role of the epigenome alongside epigenome-targeting treatments develops, we look forward to the advances made in genomic medicine and the field of psychiatry.

## References

[1] Nestler EJ Hyman SE. Animal models of neuropsychiatric disorders. Nat Neurosci. (2010) 13:1161–9. doi: 10.1038/nn.2647, PMID: 20877280 PMC3750731

[2] Turecki G Meaney MJ. Effects of the social environment and stress on glucocorticoid receptor gene methylation: A systematic review. Biol Psychiatry. (2016) 79:87–96. doi: 10.1016/j.biopsych.2014.11.022, PMID: 25687413 PMC4466091

[3] Schuebel K Gitik M Domschke K Goldman D. Making sense of epigenetics. IJNPPY. (2016) 19:pyw058. doi: 10.1093/ijnp/pyw058, PMID: 27312741 PMC5137275

[4] Qureshi IA Mehler MF. Epigenetic mechanisms underlying human epileptic disorders and the process of epileptogenesis. Neurobiol Disease. (2010) 39:53–60. doi: 10.1016/j.nbd.2010.02.005, PMID: 20188170 PMC2874104

[5] Klengel T Binder EB. Epigenetics of stress-related psychiatric disorders and gene × Environment interactions. Neuron. (2015) 86:1343–57. doi: 10.1016/j.neuron.2015.05.036, PMID: 26087162

[6] Peña CJ Nestler EJ Bagot RC. Environmental programming of susceptibility and resilience to stress in adulthood in male mice. Front Behav Neurosci. (2019) 13:40. doi: 10.3389/fnbeh.2019.00040, PMID: 30881296 PMC6405694

[7] Jaenisch R Bird A. Epigenetic regulation of gene expression: how the genome integrates intrinsic and environmental signals. Nat Genet. (2003) 33:245–54. doi: 10.1038/ng1089, PMID: 12610534

[8] Moore LD Le T Fan G. DNA methylation and its basic function. Neuropsychopharmacol. (2013) 38:23–38. doi: 10.1038/npp.2012.112, PMID: 22781841 PMC3521964

[9] Bird A. DNA methylation patterns and epigenetic memory. Genes Dev. (2002) 16:6–21. doi: 10.1101/gad.947102, PMID: 11782440

[10] McGowan PO Sasaki A D'Alessio AC Dymov S Labonté B Szyf M. Epigenetic regulation of the glucocorticoid receptor in human brain associates with childhood abuse. Nat Neurosci. (2009) 12:342–8. doi: 10.1038/nn.2270, PMID: 19234457 PMC2944040

[11] Cedar H Bergman Y. Linking DNA methylation and histone modification: patterns and paradigms. Nat Rev Genet. (2009) 10:295–304. doi: 10.1038/nrg2540, PMID: 19308066

[12] Feng J Nestler EJ. Epigenetic mechanisms of drug addiction. Curr Opin Neurobiology. (2013) 23:521–8. doi: 10.1016/j.conb.2013.01.001, PMID: 23374537 PMC3644540

[13] Ferrer A Labad J Salvat-Pujol N Barrachina M Costas J Urretavizcaya M. BDNF genetic variants and methylation: effects on cognition in major depressive disorder. Transl Psychiatry. (2019) 9:265. doi: 10.1038/s41398-019-0601-8, PMID: 31636250 PMC6803763

[14] Perroud N Paoloni-Giacobino A Prada P Olié E Salzmann A Nicastro R. Increased methylation of glucocorticoid receptor gene (NR3C1) in adults with a history of childhood maltreatment: a link with the severity and type of trauma. Transl Psychiatry. (2011) 1:e59–9. doi: 10.1038/tp.2011.60, PMID: 22832351 PMC3309499

[15] Zhou J Zhou D Yan T Chen W Xie H Xiong Y. Association between CpG island DNA methylation in the promoter region of *RELN* and positive and negative types of schizophrenia. J Int Med Res. (2022) 50:3000605221100345. doi: 10.1177/03000605221100345, PMID: 35638503 PMC9160895

[16] Dell׳Osso B D׳Addario C Palazzo MC Benatti B Camuri G Galimberti D. Epigenetic modulation of BDNF gene: Differences in DNA methylation between unipolar and bipolar patients. J Affect Disord. (2014) 166:330–3. doi: 10.1016/j.jad.2014.05.020, PMID: 25012449

[17] Schröter K Brum M Brunkhorst-Kanaan N Tole F Ziegler C Domschke K. Longitudinal multi-level biomarker analysis of BDNF in major depression and bipolar disorder. Eur Arch Psychiatry Clin Neurosci. (2020) 270:169–81. doi: 10.1007/s00406-019-01007-y, PMID: 30929061

[18] Nagarajan R Hogart A Gwye Y Martin MR LaSalle JM. Reduced meCP2 expression is frequent in autism frontal cortex and correlates with aberrant MECP2 promoter methylation. Epigenetics. (2006) 1:172–82. doi: 10.4161/epi.1.4.3514, PMID: 17486179 PMC1866172

[19] Nielsen DA Yuferov V Hamon S Jackson C Ho A Ott J. Increased OPRM1 DNA methylation in lymphocytes of methadone-maintained former heroin addicts. Neuropsychopharmacol. (2009) 34:867–73. doi: 10.1038/npp.2008.108, PMID: 18650805 PMC2778040

[20] Zhang H Herman AI Kranzler HR Anton RF Simen AA Gelernter J. Hypermethylation of OPRM1 promoter region in European Americans with alcohol dependence. J Hum Genet. (2012) 57:670–5. doi: 10.1038/jhg.2012.98, PMID: 22914673 PMC3481015

[21] Kouzarides T. Chromatin modifications and their function. Cell. (2007) 128:693–705. doi: 10.1016/j.cell.2007.02.005, PMID: 17320507

[22] Renthal W Nestler EJ. Histone acetylation in drug addiction. Semin Cell Dev Biol. (2009) 20:387–94. doi: 10.1016/j.semcdb.2009.01.005, PMID: 19560043 PMC2704458

[23] Xia C Tao Y Li M Che T Qu J. Protein acetylation and deacetylation: An important regulatory modification in gene transcription (Review). Exp Ther Med. (2020) 20(4):2923–40. doi: 10.3892/etm.2020.9073, PMID: 32855658 PMC7444376

[24] Greer EL Shi Y. Histone methylation: a dynamic mark in health, disease and inheritance. Nat Rev Genet. (2012) 13:343–57. doi: 10.1038/nrg3173, PMID: 22473383 PMC4073795

[25] Rossetto D Avvakumov N Côté J. Histone phosphorylation: A chromatin modification involved in diverse nuclear events. Epigenetics. (2012) 7:1098–108. doi: 10.4161/epi.21975, PMID: 22948226 PMC3469451

[26] Du Q Luu PL Stirzaker C Clark SJ. Methyl-cpG-binding domain proteins: readers of the epigenome. Epigenomics. (2015) 7:1051–73. doi: 10.2217/epi.15.39, PMID: 25927341

[27] Grayson DR Kundakovic M Sharma RP. Is there a future for histone deacetylase inhibitors in the pharmacotherapy of psychiatric disorders? Mol Pharmacol. (2010) 77:126–35. doi: 10.1124/mol.109.061333, PMID: 19917878

[28] Covington HEIII Maze I LaPlant QC Vialou VF Ohnishi YN Berton O. Antidepressant actions of histone deacetylase inhibitors. J Neurosci. (2009) 29:11451–60. doi: 10.1523/JNEUROSCI.1758-09.2009, PMID: 19759294 PMC2775805

[29] Bartel DP. MicroRNAs. Cell. (2004) 116:281–97. doi: 10.1016/S0092-8674(04)00045-5, PMID: 14744438

[30] Krol J Loedige I Filipowicz W. The widespread regulation of microRNA biogenesis, function and decay. Nat Rev Genet. (2010) 11:597–610. doi: 10.1038/nrg2843, PMID: 20661255

[31] Im HI Kenny PJ. MicroRNAs in neuronal function and dysfunction. Trends Neurosciences. (2012) 35:325–34. doi: 10.1016/j.tins.2012.01.004, PMID: 22436491 PMC3565236

[32] The Schizophrenia Psychiatric Genome-Wide Association Study (GWAS) Consortium. Genome-wide association study identifies five new schizophrenia loci. Nat Genet. (2011) 43:969–76. doi: 10.1038/ng.940, PMID: 21926974 PMC3303194

[33] Ripke S Sanders AR Kendler KS Levinson DF Sklar P Holmans PA. MiR-137: an important player in neural development and neoplastic transformation. Mol Psychiatry. (2017) 22:44–55. doi: 10.1038/mp.2016.150, PMID: 27620842 PMC5414082

[34] He C Wang Q Fan D Liu X Bai Y Zhang H. MicroRNA-124 influenced depressive symptoms via large-scale brain connectivity in major depressive disorder patients. Asian J Psychiatry. (2024) 95:104025. doi: 10.1016/j.ajp.2024.104025, PMID: 38522164

[35] Fang Y Qiu Q Zhang S Sun L Li G Xiao S. Changes in miRNA-132 and miR-124 levels in non-treated and citalopram-treated patients with depression. J Affect Disord. (2018) 227:745–51. doi: 10.1016/j.jad.2017.11.090, PMID: 29689690

[36] Ni L Zhu Y Lv L Zhang R Xie S Zhang X. Peripheral blood miR-16-5p as a potential biomarker for distinguishing unmedicated bipolar disorder type II from major depressive disorder. J Affect Disord. (2025) 382:453–61. doi: 10.1016/j.jad.2025.04.126, PMID: 40274128

[37] Yu H-C Wu J Zhang H-X Zhang G-L Sui J Tong W-W. Alterations of miR-132 are novel diagnostic biomarkers in peripheral blood of schizophrenia patients. Prog Neuropsychopharmacol Biol Psychiatry. (2015) 63:23–9. doi: 10.1016/j.pnpbp.2015.05.007, PMID: 25985888

[38] Issler O Haramati S Paul ED Maeno H Navon I Zwang R. MicroRNA 135 is essential for chronic stress resiliency, antidepressant efficacy, and intact serotonergic activity. Neuron. (2014) 83:344–60. doi: 10.1016/j.neuron.2014.05.042, PMID: 24952960

[39] Clapier CR Iwasa J Cairns BR Peterson CL. Mechanisms of action and regulation of ATP-dependent chromatin-remodelling complexes. Nat Rev Mol Cell Biol. (2017) 18:407–22. doi: 10.1038/nrm.2017.26, PMID: 28512350 PMC8127953

[40] Hargreaves DC Crabtree GR. ATP-dependent chromatin remodeling: genetics, genomics and mechanisms. Cell Res. (2011) 21:396–420. doi: 10.1038/cr.2011.32, PMID: 21358755 PMC3110148

[41] Ho L Crabtree GR. Chromatin remodelling during development. Nature. (2010) 463:474–84. doi: 10.1038/nature08911, PMID: 20110991 PMC3060774

[42] Neale BM Kou Y Liu L Ma’ayan A Samocha KE Sabo A. Patterns and rates of exonic *de novo* mutations in autism spectrum disorders. Nature. (2012) 485:242–5. doi: 10.1038/nature11011, PMID: 22495311 PMC3613847

[43] Labonté B Suderman M Maussion G Navaro L Yerko V Mahar I. Genome-wide epigenetic regulation by early-life trauma. Arch Gen Psychiatry. (2012) 69(7):722–31. doi: 10.1001/archgenpsychiatry.2011.2287, PMID: 22752237 PMC4991944

[44] Corces MR Buenrostro JD Wu B Greenside PG Chan SM Koenig JL. Lineage-specific and single-cell chromatin accessibility charts human hematopoiesis and leukemia evolution. Nat Genet. (2016) 48:1193–203. doi: 10.1038/ng.3646, PMID: 27526324 PMC5042844

[45] Kim SS Truong B Jagadeesh K Dey KK Shen AZ Raychaudhuri S. Leveraging single-cell ATAC-seq and RNA-seq to identify disease-critical fetal and adult brain cell types. Nat Commun. (2024) 15:563. doi: 10.1038/s41467-024-44742-0, PMID: 38233398 PMC10794712

[46] Bryois J Garrett ME Song L Safi A Giusti-Rodriguez P Johnson GD. Evaluation of chromatin accessibility in prefrontal cortex of schizophrenia cases and controls. Genomics. (2017) 9(1):3121. doi: 10.1101/141986 PMC6081462 30087329

[47] Duttke SH Montilla-Perez P Chang MW Li H Chen H Carrette LLG. Glucocorticoid receptor-regulated enhancers play a central role in the gene regulatory networks underlying drug addiction. Front Neurosci. (2022) 16:858427. doi: 10.3389/fnins.2022.858427, PMID: 35651629 PMC9149415

[48] Cruz B Vozella V Carper BA Xu JC Kirson D Hirsch S. FKBP5 inhibitors modulate alcohol drinking and trauma-related behaviors in a model of comorbid post-traumatic stress and alcohol use disorder. Neuropsychopharmacol. (2023) 48:1144–54. doi: 10.1038/s41386-022-01497-w, PMID: 36396784 PMC10267127

[49] Feil R Fraga MF. Epigenetics and the environment: emerging patterns and implications. Nat Rev Genet. (2012) 13:97–109. doi: 10.1038/nrg3142, PMID: 22215131

[50] Waterland RA Jirtle RL. Transposable elements: targets for early nutritional effects on epigenetic gene regulation. Mol Cell Biol. (2003) 23:5293–300. doi: 10.1128/MCB.23.15.5293-5300.2003, PMID: 12861015 PMC165709

[51] Gomez-Pinilla F Zhuang Y Feng J Ying Z Fan G. Exercise impacts brain-derived neurotrophic factor plasticity by engaging mechanisms of epigenetic regulation. Eur J Neurosci. (2011) 33:383–90. doi: 10.1111/j.1460-9568.2010.07508.x, PMID: 21198979 PMC3256007

[52] Byun HM Baccarelli AA. Environmental exposure and mitochondrial epigenetics: study design and analytical challenges. Hum Genet. (2014) 133:247–57. doi: 10.1007/s00439-013-1417-x, PMID: 24402053 PMC4351743

[53] Kundakovic M Gudsnuk K Herbstman JB Tang D Perera FP Champagne FA. DNA methylation of BDNF as a biomarker of early-life adversity. Proc Natl Acad Sci USA. (2015) 112:6807–13. doi: 10.1073/pnas.1408355111, PMID: 25385582 PMC4460453

[54] Senut M-C Cingolani P Sen A Kruger A Shaik A Hirsch H. Epigenetics of early-life lead exposure and effects on brain development. Epigenomics. (2012) 4:665–74. doi: 10.2217/epi.12.58, PMID: 23244311 PMC3555228

[55] Krol KM Puglia MH Morris JP Connelly JJ Grossmann T. Epigenetic modification of the oxytocin receptor gene is associated with emotion processing in the infant brain. Dev Cogn Neurosci. (2019) 37:100648. doi: 10.1016/j.dcn.2019.100648, PMID: 31125951 PMC6969294

[56] Yehuda R Daskalakis NP Desarnaud F Makotkine I Lehrner AL Koch E. Epigenetic biomarkers as predictors and correlates of symptom improvement following psychotherapy in combat veterans with PTSD. Front Psychiatry. (2013) 4:118. doi: 10.3389/fpsyt.2013.00118, PMID: 24098286 PMC3784793

[57] Nasca C Bigio B Lee FS Young SP Kautz MM Albright A. Acetyl-L-carnitine deficiency in patients with major depressive disorder. Proc Natl Acad Sci USA. (2018) 115:8627–32. doi: 10.1073/pnas.1801609115, PMID: 30061399 PMC6112703

[58] Binder DK Scharfman HE. Brain-derived neurotrophic factor. Growth Factors. (2004) 22:123–31. doi: 10.1080/08977190410001723308, PMID: 15518235 PMC2504526

[59] Lu B Martinowich K. Cell biology of BDNF and its relevance to schizophrenia. Novartis Found Symp. (2008) 289:119–29. doi: 10.1002/9780470751251.ch10, PMID: 18497099 PMC3096549

[60] Egan MF Kojima M Callicott JH Goldberg TE Kolachana BS Bertolino A. The BDNF val66met polymorphism affects activity-dependent secretion of BDNF and human memory and hippocampal function. Cell. (2003) 112:257–69. doi: 10.1016/S0092-8674(03)00035-7, PMID: 12553913

[61] Lubin FD Roth TL Sweatt JD. Epigenetic regulation of *bdnf* gene transcription in the consolidation of fear memory. J Neurosci. (2008) 28:10576–86. doi: 10.1523/JNEUROSCI.1786-08.2008, PMID: 18923034 PMC3312036

[62] Keller S Sarchiapone M Zarrilli F Videtic A Ferraro A Carli V. Increased BDNF promoter methylation in the wernicke area of suicide subjects. Arch Gen Psychiatry. (2010) 67:258. doi: 10.1001/archgenpsychiatry.2010.9, PMID: 20194826

[63] Fuchikami M Morinobu S Segawa M Okamoto Y Yamawaki S Ozaki N. DNA methylation profiles of the brain-derived neurotrophic factor (BDNF) gene as a potent diagnostic biomarker in major depression. PloS One. (2011) 6:e23881. doi: 10.1371/journal.pone.0023881, PMID: 21912609 PMC3166055

[64] Kleimann A Kotsiari A Sperling W Gröschl M Heberlein A Kahl KG. BDNF serum levels and promoter methylation of BDNF exon I, IV and VI in depressed patients receiving electroconvulsive therapy. J Neural Transm. (2015) 122:925–8. doi: 10.1007/s00702-014-1336-6, PMID: 25387785

[65] Simões Fernandes B Gama CS Ceresér KM Yatham LN Fries GR Colpo G. Brain-derived neurotrophic factor as a state-marker of mood episodes in bipolar disorders: A systematic review and meta-regression analysis. J Psychiatr Res. (2011) 45:995–1004. doi: 10.1016/j.jpsychires.2011.03.002, PMID: 21550050

[66] Botella-López A Burgaya F Gavín R García-Ayllón MS Gómez-Tortosa E Peña-Casanova J. Reelin expression and glycosylation patterns are altered in Alzheimer’s disease. Proc Natl Acad Sci USA. (2006) 103:5573–8. doi: 10.1073/pnas.0601279103, PMID: 16567613 PMC1414634

[67] Kouter K Zupanc T Videtič Paska A. Genome-wide DNA methylation in suicide victims revealing impact on gene expression. J Affect Disord. (2019) 253:419–25. doi: 10.1016/j.jad.2019.04.077, PMID: 31103807

[68] Dwivedi Y Rizavi HS Conley RR Roberts RC Tamminga CA Pandey GN. Altered gene expression of brain-derived neurotrophic factor and receptor tyrosine kinase B in postmortem brain of suicide subjects. Arch Gen Psychiatry. (2003) 60:804. doi: 10.1001/archpsyc.60.8.804, PMID: 12912764

[69] Tsankova NM Kumar A Nestler EJ. Histone Modifications at Gene Promoter Regions in Rat Hippocampus after Acute and Chronic Electroconvulsive Seizures. J Neurosci. (2004) 24:5603–10. doi: 10.1523/JNEUROSCI.0589-04.2004, PMID: 15201333 PMC6729334

[70] Schroeder FA Lin CL Crusio WE Akbarian S. Antidepressant-like effects of the histone deacetylase inhibitor, sodium butyrate, in the mouse. Biol Psychiatry. (2007) 62:55–64. doi: 10.1016/j.biopsych.2006.06.036, PMID: 16945350

[71] Gao J Wang W-Y Mao Y-W Gräff J Guan J-S Pan L. A novel pathway regulates memory and plasticity via SIRT1 and miR-134. Nature. (2010) 466:1105–9. doi: 10.1038/nature09271, PMID: 20622856 PMC2928875

[72] Männistö PT Kaakkola S. Catechol-O-methyltransferase (COMT): biochemistry, molecular biology, pharmacology, and clinical efficacy of the new selective COMT inhibitors. Pharmacol Rev. (1999) 51:593–628. doi: 10.1016/S0031-6997(24)01423-6, PMID: 10581325

[73] Chen J Lipska BK Halim N Ma QD Matsumoto M Melhem S. Functional analysis of genetic variation in catechol-O-methyltransferase (COMT): effects on mRNA, protein, and enzyme activity in postmortem human brain. Am J Hum Genet. (2004) 75:807–21. doi: 10.1086/425589, PMID: 15457404 PMC1182110

[74] Abdolmaleky HM Cheng K-H Faraone SV Wilcox M Glatt SJ Gao F. Hypomethylation of MB-COMT promoter is a major risk factor for schizophrenia and bipolar disorder. Hum Mol Genet. (2006) 15:3132–45. doi: 10.1093/hmg/ddl253, PMID: 16984965 PMC2799943

[75] Drabe M Rullmann M Luthardt J Boettcher Y Regenthal R Ploetz T. Serotonin transporter gene promoter methylation status correlates with *in vivo* prefrontal 5-HTT availability and reward function in human obesity. Transl Psychiatry. (2017) 7:e1167–7. doi: 10.1038/tp.2017.133, PMID: 28675387 PMC5538116

[76] Nohesara S Ghadirivasfi M Mostafavi S Eskandari M-R Ahmadkhaniha H Thiagalingam S. DNA hypomethylation of MB-COMT promoter in the DNA derived from saliva in schizophrenia and bipolar disorder. J Psychiatr Res. (2011) 45:1432–8. doi: 10.1016/j.jpsychires.2011.06.013, PMID: 21820670

[77] Lachman HM Papolos DF Saito T Yu YM Szumlanski CL Weinshilboum RM. Human catechol-O-methyltransferase pharmacogenetics: description of a functional polymorphism and its potential application to neuropsychiatric disorders. Pharmacogenetics. (1996) 6:243–50. doi: 10.1097/00008571-199606000-00007, PMID: 8807664

[78] Wiegand A Blickle A Brückmann C Weller S Nieratschker V Plewnia C. Dynamic DNA methylation changes in the COMT gene promoter region in response to mental stress and its modulation by transcranial direct current stimulation. Biomolecules. (2021) 11:1726. doi: 10.3390/biom11111726, PMID: 34827724 PMC8615564

[79] Norrholm SD Jovanovic T Smith AK Binder E Klengel T Conneely K. Differential genetic and epigenetic regulation of catechol-O-methyltransferase is associated with impaired fear inhibition in posttraumatic stress disorder. Front Behav Neurosci. (2013) 7:30. doi: 10.3389/fnbeh.2013.00030, PMID: 23596403 PMC3622057

[80] Xu Q Ma JZ Payne TJ Li MD. Determination of methylated cpG sites in the promoter region of catechol-O-methyltransferase (COMT) and their involvement in the etiology of tobacco smoking. Front Psychiatry. (2010) 1:16. doi: 10.3389/fpsyt.2010.00016, PMID: 21423427 PMC3059640

[81] Van Der Knaap LJ Schaefer JM Franken IHA Verhulst FC Van Oort FVA Riese H. Catechol-O-methyltransferase gene methylation and substance use in adolescents: the TRAILS study. Genes Brain Behavior. (2014) 13:618–25. doi: 10.1111/gbb.12147, PMID: 24902721

[82] Jaffe AE Gao Y Deep-Soboslay A Tao R Hyde TM Weinberger DR. Mapping DNA methylation across development, genotype and schizophrenia in the human frontal cortex. Nat Neurosci. (2016) 19:40–7. doi: 10.1038/nn.4181, PMID: 26619358 PMC4783176

[83] Zannas AS Wiechmann T Gassen NC Binder EB. Gene–stress–epigenetic regulation of FKBP5: clinical and translational implications. Neuropsychopharmacol. (2016) 41:261–74. doi: 10.1038/npp.2015.235, PMID: 26250598 PMC4677131

[84] Binder EB. The role of FKBP5, a co-chaperone of the glucocorticoid receptor in the pathogenesis and therapy of affective and anxiety disorders. Psychoneuroendocrinology. (2009) 34:S186–95. doi: 10.1016/j.psyneuen.2009.05.021, PMID: 19560279

[85] Klengel T Mehta D Anacker C Rex-Haffner M Pruessner JC Pariante CM. Allele-specific FKBP5 DNA demethylation mediates gene–childhood trauma interactions. Nat Neurosci. (2013) 16:33–41. doi: 10.1038/nn.3275, PMID: 23201972 PMC4136922

[86] Klengel T Binder EB. FKBP5 allele-specific epigenetic modification in gene by environment interaction. Neuropsychopharmacol. (2015) 40:244–6. doi: 10.1038/npp.2014.208, PMID: 25482174 PMC4262902

[87] Caradonna SG Paul MR Marrocco J. An allostatic epigenetic memory on chromatin footprints after double-hit acute stress. Neurobiol Stress. (2022) 20:100475. doi: 10.1016/j.ynstr.2022.100475, PMID: 36032404 PMC9400173

[88] Maurel OM Torrisi SA Barbagallo C Purrello M Salomone S Drago F. Dysregulation of miR-15a-5p, miR-497a-5p and miR-511-5p Is Associated with Modulation of BDNF and FKBP5 in Brain Areas of PTSD-Related Susceptible and Resilient Mice. Int J Mol Sci. (2021) 22:5157. doi: 10.3390/ijms22105157, PMID: 34068160 PMC8153003

[89] Höhne N Poidinger M Merz F Pfister H Brückl T Zimmermann P. FKBP5 genotype-dependent DNA methylation and mRNA regulation after psychosocial stress in remitted depression and healthy controls. Int J Neuropsychopharmacol. (2015) 18:pyu087–7. doi: 10.1093/ijnp/pyu087, PMID: 25522420 PMC4360217

[90] Menke A Arloth J Pütz B Weber P Klengel T Mehta D. Dexamethasone stimulated gene expression in peripheral blood is a sensitive marker for glucocorticoid receptor resistance in depressed patients. Neuropsychopharmacol. (2012) 37:1455–64. doi: 10.1038/npp.2011.331, PMID: 22237309 PMC3327850

[91] Seo JH Kim ST Jeon S Kang JI Kim SJ. Sex-dependent association of DNA methylation of HPA axis-related gene FKBP5 with obsessive-compulsive disorder. Psychoneuroendocrinology. (2023) 158:106404. doi: 10.1016/j.psyneuen.2023.106404, PMID: 37769537

[92] Zou Z Huang Y Maes M Wang J He Y Min W. Effects of antidepressant on FKBP51 mRNA expression and neuroendocrine hormones in patients with panic disorder. BMC Psychiatry. (2024) 24:269. doi: 10.1186/s12888-024-05704-4, PMID: 38600448 PMC11005249

[93] Roberts S Keers R Breen G Coleman JRI Jöhren P Kepa A. DNA methylation of FKBP5 and response to exposure-based psychological therapy. Am J Med Genet Pt B. (2019) 180:150–8. doi: 10.1002/ajmg.b.32650, PMID: 30334356 PMC6600698

[94] Bishop JR Lee AM Mills LJ Thuras PD Eum S Clancy D. Methylation of FKBP5 and SLC6A4 in relation to treatment response to mindfulness based stress reduction for posttraumatic stress disorder. Front Psychiatry. (2018) 9:418. doi: 10.3389/fpsyt.2018.00418, PMID: 30279666 PMC6153325

[95] Oakley RH Cidlowski JA. The biology of the glucocorticoid receptor: New signaling mechanisms in health and disease. J Allergy Clin Immunol. (2013) 132:1033–44. doi: 10.1016/j.jaci.2013.09.007, PMID: 24084075 PMC4084612

[96] McEwen BS Morrison JH. The brain on stress: vulnerability and plasticity of the prefrontal cortex over the life course. Neuron. (2013) 79:16–29. doi: 10.1016/j.neuron.2013.06.028, PMID: 23849196 PMC3753223

[97] Palma-Gudiel H Córdova-Palomera A Leza JC Fañanás L. Glucocorticoid receptor gene (NR3C1) methylation processes as mediators of early adversity in stress-related disorders causality: A critical review. Neurosci Biobehav Rev. (2015) 55:520–35. doi: 10.1016/j.neubiorev.2015.05.016, PMID: 26073068

[98] Tyrka AR Price LH Marsit C Walters OC Carpenter LL. Childhood adversity and epigenetic modulation of the leukocyte glucocorticoid receptor: preliminary findings in healthy adults. PloS One. (2012) 7:e30148. doi: 10.1371/journal.pone.0030148, PMID: 22295073 PMC3266256

[99] Tyrka AR Price LH Marsit C Walters OC Carpenter LL. Child abuse, depression, and methylation in genes involved with stress, neural plasticity, and brain circuitry. J Am Acad Child Adolesc Psychiatry. (2014) 53:417–424.e5. doi: 10.1016/j.jaac.2013.12.025, PMID: 24655651 PMC4126411

[100] Lesch KP Bengel D Heils A Sabol SZ Greenberg BD Petri S. Association of anxiety-related traits with a polymorphism in the serotonin transporter gene regulatory region. Science. (1996) 274:1527–31. doi: 10.1126/science.274.5292.1527, PMID: 8929413

[101] Canli T Lesch KP. Long story short: the serotonin transporter in emotion regulation and social cognition. Nat Neurosci. (2007) 10:1103–9. doi: 10.1038/nn1964, PMID: 17726476

[102] Hariri AR Mattay VS Tessitore A Kolachana B Fera F Goldman D. Serotonin transporter genetic variation and the response of the human amygdala. Science. (2002) 297:400–3. doi: 10.1126/science.1071829, PMID: 12130784

[103] Booij L Szyf M Carballedo A Frey E-M Morris D Dymov S. DNA methylation of the serotonin transporter gene in peripheral cells and stress-related changes in hippocampal volume: A study in depressed patients and healthy controls. PloS One. (2015) 10:e0119061. doi: 10.1371/journal.pone.0119061, PMID: 25781010 PMC4363605

[104] Van IJzendoorn MH Caspers K Bakermans-Kranenburg MJ Beach SRH Philibert R. Methylation matters: interaction between methylation density and serotonin transporter genotype predicts unresolved loss or trauma. Biol Psychiatry. (2010) 68:405–7. doi: 10.1016/j.biopsych.2010.05.008, PMID: 20591416 PMC2921470

[105] Kang H-J Kim J-M Stewart R Kim S-Y Bae K-Y Kim S-W. Association of SLC6A4 methylation with early adversity, characteristics and outcomes in depression. Prog Neuropsychopharmacol Biol Psychiatry. (2013) 44:23–8. doi: 10.1016/j.pnpbp.2013.01.006, PMID: 23333376

[106] Okada S Morinobu S Fuchikami M Segawa M Yokomaku K Kataoka T. The potential of SLC6A4 gene methylation analysis for the diagnosis and treatment of major depression. J Psychiatr Res. (2014) 53:47–53. doi: 10.1016/j.jpsychires.2014.02.002, PMID: 24657235

[107] Furmark T Appel L Henningsson S Ahs F Faria V Linnman C. A link between serotonin-related gene polymorphisms, amygdala activity, and placebo-induced relief from social anxiety. J Neurosci. (2008) 28:13066–74. doi: 10.1523/JNEUROSCI.2534-08.2008, PMID: 19052197 PMC6671592

[108] Mooney MA Ryabinin P Wilmot B Bhatt P Mill J Nigg JT. Large epigenome-wide association study of childhood ADHD identifies peripheral DNA methylation associated with disease and polygenic risk burden. Transl Psychiatry. (2020) 10:8. doi: 10.1038/s41398-020-0710-4, PMID: 32066674 PMC7026179

[109] Beaulieu JM Gainetdinov RR. The physiology, signaling, and pharmacology of dopamine receptors. Pharmacol Rev. (2011) 63:182–217. doi: 10.1124/pr.110.002642, PMID: 21303898

[110] Missale C Nash SR Robinson SW Jaber M Caron MG. Dopamine receptors: from structure to function. Physiol Rev. (1998) 78:189–225. doi: 10.1152/physrev.1998.78.1.189, PMID: 9457173

[111] Volkow ND Wang GJ Fowler JS Telang F. Overlapping neuronal circuits in addiction and obesity: evidence of systems pathology. Phil Trans R Soc B. (2008) 363:3191–200. doi: 10.1098/rstb.2008.0107, PMID: 18640912 PMC2607335

[112] Usiello A Paladini AA Gargiulo A Di Benedetto M Florio E Di Cunto F. Distinct functions of the two isoforms of dopamine D2 receptors. Nature. (2000) 408:199–203. doi: 10.1038/35041572, PMID: 11089973

[113] Abdolmaleky HM Smith CL Faraone SV Shafa R Stone W Glatt SJ. Methylomics in psychiatry: Modulation of gene–environment interactions may be through DNA methylation. Am J Med Genet Pt B. (2004) 127B:51–9. doi: 10.1002/ajmg.b.20142, PMID: 15108180

[114] Viana J Hannon E Dempster E Pidsley R Macdonald R Knox O. Schizophrenia-associated methylomic variation: molecular signatures of disease and polygenic risk burden across multiple brain regions. Hum Mol Genet. (2016) 26(1):210–25. doi: 10.1093/hmg/ddw373, PMID: 28011714 PMC5351932

[115] Shi S Leites C He D Schwartz D Moy W Shi J. MicroRNA-9 and microRNA-326 regulate human dopamine D2 receptor expression, and the microRNA-mediated expression regulation is altered by a genetic variant. J Biol Chem. (2014) 289:13434–44. doi: 10.1074/jbc.M113.535203, PMID: 24675081 PMC4036351

[116] Howes OD Kapur S. The dopamine hypothesis of schizophrenia: version III–the final common pathway. Schizophr Bulletin. (2009) 35:549–62. doi: 10.1093/schbul/sbp006, PMID: 19325164 PMC2669582

[117] Zhang TY Meaney MJ. Epigenetics and the environmental regulation of the genome and its function. Annu Rev Psychol. (2010) 61:439–66. doi: 10.1146/annurev.psych.60.110707.163625, PMID: 19958180

[118] Nielsen DA Utrankar A Reyes JA Simons DD Kosten TR. Epigenetics of drug abuse: predisposition or response. Pharmacogenomics. (2012) 13:1149–60. doi: 10.2217/pgs.12.94, PMID: 22909205 PMC3463407

[119] Suderman M Borghol N Pappas JJ Pinto Pereira SM Pembrey M Hertzman C. Molecular genetics of attention-deficit/hyperactivity disorder: an overview. Eur Child Adolesc Psychiatry. (2010) 19:237–57. doi: 10.1007/s00787-010-0090-z, PMID: 20145962 PMC2839490

[120] Tsankova N Renthal W Kumar A Nestler EJ. Epigenetic regulation in psychiatric disorders. Nat Rev Neurosci. (2007) 8:355–67. doi: 10.1038/nrn2132, PMID: 17453016

[121] Nestler EJ. Molecular mechanisms of drug addiction. Neuropharmacology. (2004) 47:24–32. doi: 10.1016/j.neuropharm.2004.06.031, PMID: 15464123

[122] Mellios N Sur M. The emerging role of microRNAs in schizophrenia and autism spectrum disorders. Front Psychiatry. (2012) 3:39. doi: 10.3389/fpsyt.2012.00039, PMID: 22539927 PMC3336189

[123] Bocchio-Chiavetto L Maffioletti E Bettinsoli P Giovannini C Bignotti S Tardito D. Blood microRNA changes in depressed patients during antidepressant treatment. Eur Neuropsychopharmacol. (2013) 23:602–11. doi: 10.1016/j.euroneuro.2012.06.013, PMID: 22925464

[124] Rahman MF McGowan PO. Cell-type-specific epigenetic effects of early life stress on the brain. Transl Psychiatry. (2022) 12:326. doi: 10.1038/s41398-022-02076-9, PMID: 35948532 PMC9365848

[125] Liu XS Wu H Ji X Stelzer Y Wu X Czauderna S. Editing DNA methylation in the mammalian genome. Cell. (2016) 167:233–247.e17. doi: 10.1016/j.cell.2016.08.056, PMID: 27662091 PMC5062609

[126] Thakore PI D'Ippolito AM Song L Safi A Shivakumar NK Kabadi AM. Highly specific epigenome editing by CRISPR-Cas9 repressors for silencing of distal regulatory elements. Nat Methods. (2015) 12:1143–9. doi: 10.1038/nmeth.3630, PMID: 26501517 PMC4666778

[127] Provençal N Binder EB. The effects of early life stress on the epigenome: From the womb to adulthood and even before. Exp Neurology. (2015) 268:10–20. doi: 10.1016/j.expneurol.2014.09.001, PMID: 25218020

[128] Cicchetti D Handley ED. Child maltreatment and the development of substance use and disorder. Neurobiol Stress. (2019) 10:100144. doi: 10.1016/j.ynstr.2018.100144, PMID: 30937350 PMC6430405

[129] Mohammadi S Beh-Pajooh A Ahmadimanesh M Amini M Ghazi-Khansari M Moallem SA. Evaluation of DNA methylation in *BDNF*, *SLC6A4*, *NR3C1* and *FKBP5* before and after treatment with selective serotonin-reuptake inhibitor in major depressive disorder. Epigenomics. (2022) 14:1269–80. doi: 10.2217/epi-2022-0246, PMID: 36377555

[130] Grayson DR Jia X Chen Y Sharma RP Mitchell CP Guidotti A. Reelin promoter hypermethylation in schizophrenia. Proc Natl Acad Sci USA. (2005) 102:9341–6. doi: 10.1073/pnas.0503736102, PMID: 15961543 PMC1166626

